# Cytoplasmic DNA sensing boosts CD4^+^ T cell metabolism for inflammatory induction

**DOI:** 10.1093/lifemedi/lnad021

**Published:** 2023-06-13

**Authors:** Jialin Ye, Jiemeng Fu, Hui Hou, Yan Wang, Wei Deng, Shumeng Hao, Yifei Pei, Jing Xu, Mingyue Zheng, Yichuan Xiao

**Affiliations:** CAS Key Laboratory of Tissue Microenvironment and Tumor, Shanghai Institute of Nutrition and Health, University of Chinese Academy of Sciences, Chinese Academy of Sciences, Shanghai 200031, China; CAS Key Laboratory of Tissue Microenvironment and Tumor, Shanghai Institute of Nutrition and Health, University of Chinese Academy of Sciences, Chinese Academy of Sciences, Shanghai 200031, China; Drug Discovery and Design Center, State Key Laboratory of Drug Research, Shanghai Institute of Materia Medica, University of Chinese Academy of Sciences, Chinese Academy of Sciences, Shanghai 201203, China; CAS Key Laboratory of Tissue Microenvironment and Tumor, Shanghai Institute of Nutrition and Health, University of Chinese Academy of Sciences, Chinese Academy of Sciences, Shanghai 200031, China; CAS Key Laboratory of Tissue Microenvironment and Tumor, Shanghai Institute of Nutrition and Health, University of Chinese Academy of Sciences, Chinese Academy of Sciences, Shanghai 200031, China; CAS Key Laboratory of Tissue Microenvironment and Tumor, Shanghai Institute of Nutrition and Health, University of Chinese Academy of Sciences, Chinese Academy of Sciences, Shanghai 200031, China; CAS Key Laboratory of Tissue Microenvironment and Tumor, Shanghai Institute of Nutrition and Health, University of Chinese Academy of Sciences, Chinese Academy of Sciences, Shanghai 200031, China; CAS Key Laboratory of Tissue Microenvironment and Tumor, Shanghai Institute of Nutrition and Health, University of Chinese Academy of Sciences, Chinese Academy of Sciences, Shanghai 200031, China; Drug Discovery and Design Center, State Key Laboratory of Drug Research, Shanghai Institute of Materia Medica, University of Chinese Academy of Sciences, Chinese Academy of Sciences, Shanghai 201203, China; CAS Key Laboratory of Tissue Microenvironment and Tumor, Shanghai Institute of Nutrition and Health, University of Chinese Academy of Sciences, Chinese Academy of Sciences, Shanghai 200031, China

**Keywords:** CD4^+^ T, DNA sensing, glycolysis, inflammation, autoimmunity disease

## Abstract

DNA accumulation is associated with the development of autoimmune inflammatory diseases. However, the pathological role and underlying mechanism of cytoplasmic DNA accumulation in CD4^+^ T cells have not been well established. Here, we show that *Trex1* deficiency-induced endogenous DNA accumulation in CD4^+^ T cells greatly promoted their induction of autoimmune inflammation in a lupus-like mouse model. Mechanistically, the accumulated DNA in CD4^+^ T cells was sensed by the KU complex, then triggered the activation of DNA-PKcs and ZAK and further facilitated the activation of AKT, which exacerbated glycolysis, thereby promoting the inflammatory responses. Accordingly, blocking the DNA sensing pathway in CD4^+^ T cells by genetic knockout of *Zak* or using our newly developed ZAK inhibitor iZAK2 attenuated all pathogenic characteristics in a lupus-like inflammation mouse model induced with *Trex1*-deficient CD4^+^ T cells. Overall, our study demonstrated a causal link between DNA-sensing and metabolic reprogramming in CD4^+^ T cells for inflammatory induction and suggested inhibition of the DNA sensing pathway may be a potential therapy for the treatment of inflammatory diseases.

## Introduction

Immune system evolves multiple mechanisms for controlling self-reactivity to maintain homeostasis, defects in which can cause pathogenic autoimmunity, defined as ‘autoimmune diseases’ [1]. Emerging evidences have revealed that self-DNA accumulation gives rise to autoimmune diseases in mutant mice harboring DNA metabolism and in patients with Aicardi–Goutieres Syndrome (AGS) and systemic lupus erythematosus (SLE) [1–7]. In addition, there is much progress in defining pathogenic mechanisms associated with the nucleic-acid sensors in innate immune cells, especially the cGAS-STING pathway [8–10]. For example, ablation of Trex1, an exonuclease degrading DNA in the cytoplasm, renders mice to develop an autoinflammatory phenotype accompanied by elevated expression of interferon (IFN)-stimulated genes (ISGs), which can be reversed by inhibition of cGAS or STING [9, 11]. Defects arising from RNase H2 are linked to lethal autoimmune diseases including AGS and SLE, with massive IFNs in a cGAS-STING-dependent manner [12, 13].

Since the discovery of high levels of cell-free DNA (cfDNA) in SLE patients in 1966 [14], cfDNA has been considered a potential biomarker for autoimmunity disease. There are numerous studies revealing the mechanisms of cfDNA release in SLE, which mainly include cell death processes and active release [15]. SLE is a disease characterized by accelerated apoptosis and impaired clearance of apoptotic cells, and shows evidence of cfDNA with an apoptosis-like size distribution pattern [16]. Studies in SLE patients indicated that ATP depletion resulted in the sensitization of CD4^+^ T cells to undergo necrosis, thus enabling the release of cfDNA. In addition, neutrophil extracellular traps, a structure in which the extracellular chromatin fibrils entangle microbes, are also found to deposit under SLE [17]. Cells could also secret DNA in the form of microparticles, the frequency of which is higher in the circulation of SLE patients [18]. And extracellular DNA plays an important role in the pathogenesis of SLE. For example, Toll-like receptor 9, expressed mainly within plasmacytoid dendritic cells, recognizes DNA and leads to type Ⅰ IFN production, which has been closely associated with SLE in human and mouse models [19]. However, the involvement of DNA sensing in the adaptive immune cells in accumulated-DNA-driving autoimmune diseases has not been clearly clarified. Whats more, the mechanism behind accumulated-DNA-driving autoimmune diseases is essential to be elaborate.

Our previous study showed that cytoplasmic DNA sensed by the KU complex in CD4^+^ T cells potentiates proliferation and exacerbates autoimmune inflammatory pathology in aged mice through activation of the kinase ZAK, suggesting a potential role of DNA sensing in CD4^+^ T cells during SLE pathogenesis [20].

T cells responding to stimuli exhibit rapid proliferation and differentiation with higher demand for energy and biosynthesis, which requires metabolic reprogramming. Quiescent T cells are characterized by importing small amounts of glucose, fatty acids, and amino acids to fuel the tricarboxylic acid cycle and oxidative phosphorylation (OXPHOS) metabolism. Following activation, the proliferating or autoreactive T cells initiate metabolic reprogramming, including increasing mitochondrial biogenesis, promoting glutaminolysis, and using primarily glycolysis to support anabolic pathway [21–25]. The role of glycolysis in activated T cells has been well demonstrated. Deficiency of *Glut1* in CD4^+^ T cells attenuates glucose uptake, glycolysis, and further prevents proliferation and differentiation [26]. Ablation of *Glut3* in T cells prevented Th17-cell-mediated immune response by controlling the production of acetyl-CoA and histone acetylation of cytokines [27]. SLE is an autoimmune disease in which autoreactive CD4^+^ T cells play an essential role. Several studies have demonstrated elevated glycolysis and OXPHOS in CD4^+^ T cells in SLE [28–30]. As mentioned above, deficiency of self-DNA clearance is responsible for SLE, however, the link between self-DNA accumulation and metabolism reprogramming in CD4^+^ T cells has not been investigated.

Here, we showed that the overactivated DNA sensing through the KU-ZAK system in *Trex1*^–/–^CD4^+^ T cells greatly enhanced the glycolysis, thereby leading to sever pathogenic symptoms in an inducible lupus-like inflammation model, which is a novel mechanism for accumulated DNA in pathogenesis in SLE. Accordingly, targeting the KU-mediated DNA sensing pathway by using the KU or ZAK inhibitor suppressed CD4^+^ T cell glycolysis and thus alleviated autoimmune inflammation.

## Results

### DNA accumulation in CD4^+^ T cells promotes glycolysis and inflammatory attack

**Figure 1.**
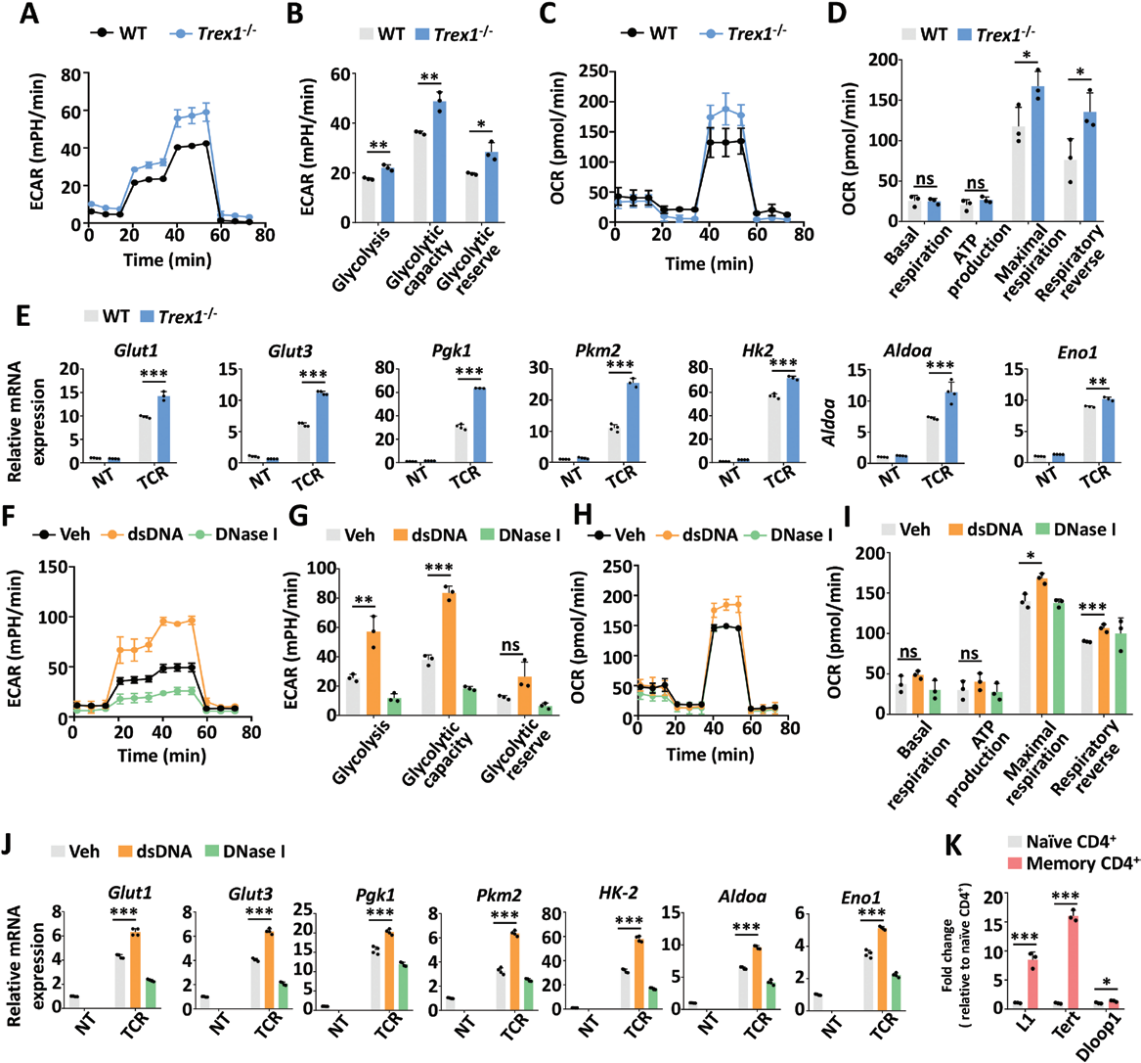
DNA accumulation boosted glycolysis and OXPHOS in CD4^+^ T cells. (A–D) Extracellular acidification rate (ECAR) and oxygen consumption rate (OCR) measurements of WT and *Trex1*^–/–^CD4^+^ T cells upon TCR stimulation (α-CD3/28, 1 μg/mL) for 24 h. (B, D) The statistical results are presented as a bar graph. (E) The transcriptional levels of glycolysis-related genes in WT and *Trex1*^–/–^ CD4^+^ T cells *ex vivo* and under TCR stimulation (α-CD3/28, 1 μg/mL, TCR) for 36 h. (F–I) ECAR and OCR measurements of CD4^+^ T cells treated with dsDNA or DNase Ⅰ upon TCR stimulation (α-CD3/28, 1 μg/mL) for 24 h. (G, I) The statistical results are presented as a bar graph. (J) The transcriptional levels of glycolysis-related genes in CD4^+^ T cells treated with dsDNA or DNase Ⅰ *ex vivo* and under TCR stimulation (α-CD3/28, 1 μg/mL, TCR) for 36 h. (K) qPCR analysis of cytoplasmic DNA extracted in naïve CD4^+^ (CD44^-^CD62L^+^CD4^+^) and memory CD4^+^ (CD44^+^CD62L^-^CD4^+^) T cells *ex vivo*. Statistics, two-tailed Students *t* test. Error bars represent SD. Differences were considered to be significant at *P* < 0.05 and are indicated by *, those at *P* < 0.01 are indicated by **, and those at *P* < 0.001 are indicated by ***.

To examine the role of DNA accumulation in CD4^+^ T cells, we analyzed the cellular metabolism in *Trex1*^–/–^ CD4^+^ T cells, which displayed self-DNA accumulation in the cytosol [31, 32]. *Trex1*^–/–^CD4^+^ T cells exhibited increased glycolysis and OXPHOS upon TCR activation, as measured by the extracellular acidification rate (ECAR), the oxygen consumption rate (OCR), and transcriptional levels of related genes (Fig. 1A–E). Consistently, transfection of double-stranded DNA (dsDNA) in CD4^+^ T cells promoted glycolysis and OXPHOS, which can be reversed by digesting cytoplasmic DNA through co-treating with DNase Ⅰ (Fig. 1F–J). We also observed more nuclear DNA leakage such as *L1* and *Tert*, as well as more mitochondrial DNA *Dloop1* in memory CD4^+^ T cells than in naïve CD4^+^ T cells, which support that T cell activation, with higher metabolic activity, was accompanied by increased cytoplasmic DNA contents (Fig. 1K). Previous study found both higher ECAR and OCR in naive CD4^+^ T cells in SLE, which is further amplified with age and activation [28]. And our previous study showed more dsDNA in aged or activated CD4^+^ T cells [20]. Those were consistent with our study here, showing the correlation between DNA contents and metabolism rates in CD4^+^ T cells.

**Figure 2.**
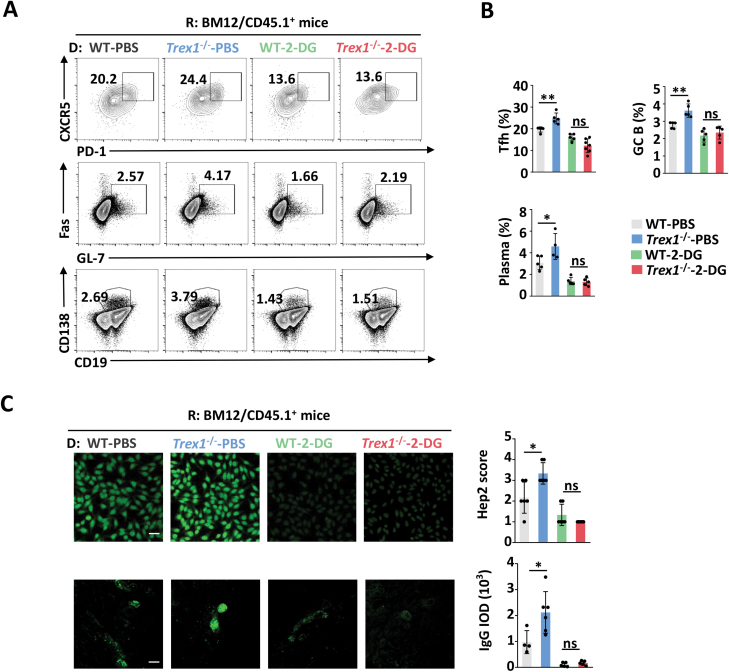
DNA accumulation in CD4^+^ T cells potentiates inflammatory attack through glycolysis. (A–C) BM12/SJL mice (recipient, R) were immunized with WT and *Trex1*^–/–^ CD4^+^ T cells (donor, D), and then intraperitoneally injected once every day with 2-DG (200 mg/kg) or PBS as control for 3 weeks. (A, B) Flow cytometric analysis of Tfh (CXCR5^+^PD1^+^CD4^+^), GC B (Fas^+^GL-7^+^CD19^+^), and plasma cells (CD138^+^CD19^lo^) in the spleen. Immunofluorescent analysis of anti-nuclear antibody (ANA) in serum (C, top) and IgG deposition in the kidney (C, bottom, scale bar: 50 μm). Data are presented as immunofluorescent images (C, left, scale bar: 50 μm) and quantification bar graphs (C, right). Statistics, two-tailed Students *t* test. Error bars represent SD. Differences were considered to be significant at *P* < 0.05 and are indicated by *, those at *P* < 0.01 are indicated by **, and those at *P* < 0.001 are indicated by ***.

In our previous work, accumulated DNA in CD4^+^ T cells promoted proliferation and pathogenic phenotypes in experimental autoimmune encephalomyelitis (EAE) [20]. Considering that glycolysis has been shown to be involved in CD4^+^ T cell activation and inflammatory response [33], we proposed that elevated glycolysis may be intermediate between DNA accumulation and T cell-mediated autoimmune diseases. To verify our hypothesis, we adoptively transferred CD4^+^ T cells from C57BL/6 into BM12 mice to establish a lupus-like inflammation mouse model [34, 35]. The results showed mice immunized with *Trex1*^-/-^ CD4^+^ developed more sever pathogenic symptoms than those transferred with WT CD4^+^ T cells, including the increased percentages of T follicular helper cells (Tfh), germinal center (GC) B cells, and plasma cells in the spleen (Fig. 2A and 2B), and more IgG deposition in the kidney as well as the higher levels of anti-nuclear antibodies (ANAs) in the serum (Fig. 2C). The administration of 2-Deoxy-D-glucose (2-DG), an inhibitor of glycolysis, clearly normalized the SLE-like pathological characteristics in *Trex1*^–/–^CD4^+^ T cells immunized BM12 mice (Fig. 2A–C). Previous evidence has demonstrated that inhibiting glycolysis using 2-DG ameliorated SLE biomarkers in the NZB/W spontaneous lupus model [28], and our results are consistent with those. Collectively, these data support the conclusion that DNA accumulation causes SLE with sever inflammatory attack for fueling glycolysis in CD4^+^ T cells.

### Accumulated DNA-fueled glycolysis is independent of cGAS-STING pathway

**Figure 3.**
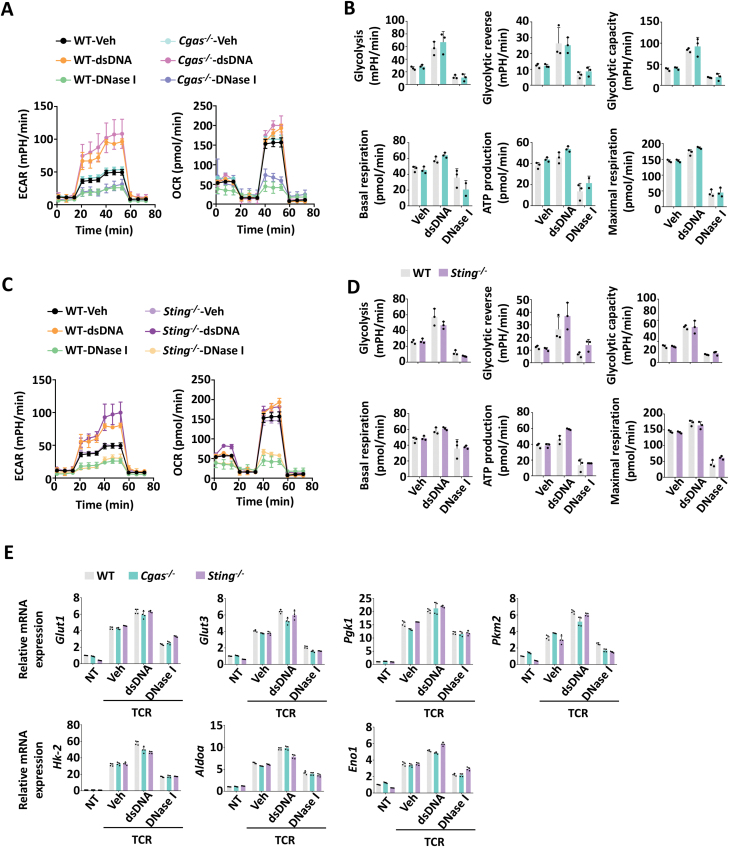
DNA-mediated glycolysis in CD4^+^ T cells is independent of the cGAS-STING pathway. (A–D) ECAR and OCR analysis of WT and *Cgas*^-/-^ or *Sting*^-/-^ CD4^+^ T cells treated with dsDNA and DNase Ⅰ under TCR activation for 24 h. (B, D) The statistical results are presented as a bar graph. (E) The transcriptional levels of glycolysis-related genes in WT, *Cgas*^-/-^ and *Sting*^-/-^ CD4^+^ T cells treated with dsDNA or DNase Ⅰ *ex vivo* and under TCR stimulation (α-CD3/28, 1 μg/mL, TCR) for 36 h. Statistics, two-tailed Students *t* test. Error bars represent SD. Differences were considered to be significant at *P* < 0.05 and are indicated by *, those at *P* < 0.01 are indicated by **, and those at *P* < 0.001 are indicated by ***.

A number of studies have revealed that a central regulator of cytosolic DNA sensing is cGAS, which is activated upon binding to dsDNA, and triggers the signaling cascade of STING to produce a battery of immune and inflammatory mediators, including type Ⅰ and Ⅲ IFNs [8, 10]. And recent works showed that the KU complex could also sense cytosolic DNA to mediate the production of IFNs in different types of human and rodent cells and promote proliferation through the activation of DNA-PKcs and ZAK in CD4^+^ T cells independent of the cGAS-STING system [20]. To clarify the underlying mechanism by which cytoplasmic DNA regulates glycolysis in CD4^+^ T cells, we first excluded the role of the cGAS-STING pathway because deletion of cGAS or STING neither affect glycolysis and OXPHOS in CD4^+^ T cells transfected with dsDNA nor in CD4^+^ T cells treated with DNase Ⅰ, which was observed using ECAR, OCR, and QPCR analysis of glycolysis-related genes (Fig. 3). Considering the low expression of cGAS in CD4^+^ T cells in our previous study [20], the conclusion that cGAS-STING system didnt mediate DNA-boosted metabolism is greatly justified.

### Blocking DNA sensing signaling in CD4^+^ T cells suppresses glycolysis and SLE

**Figure 4.**
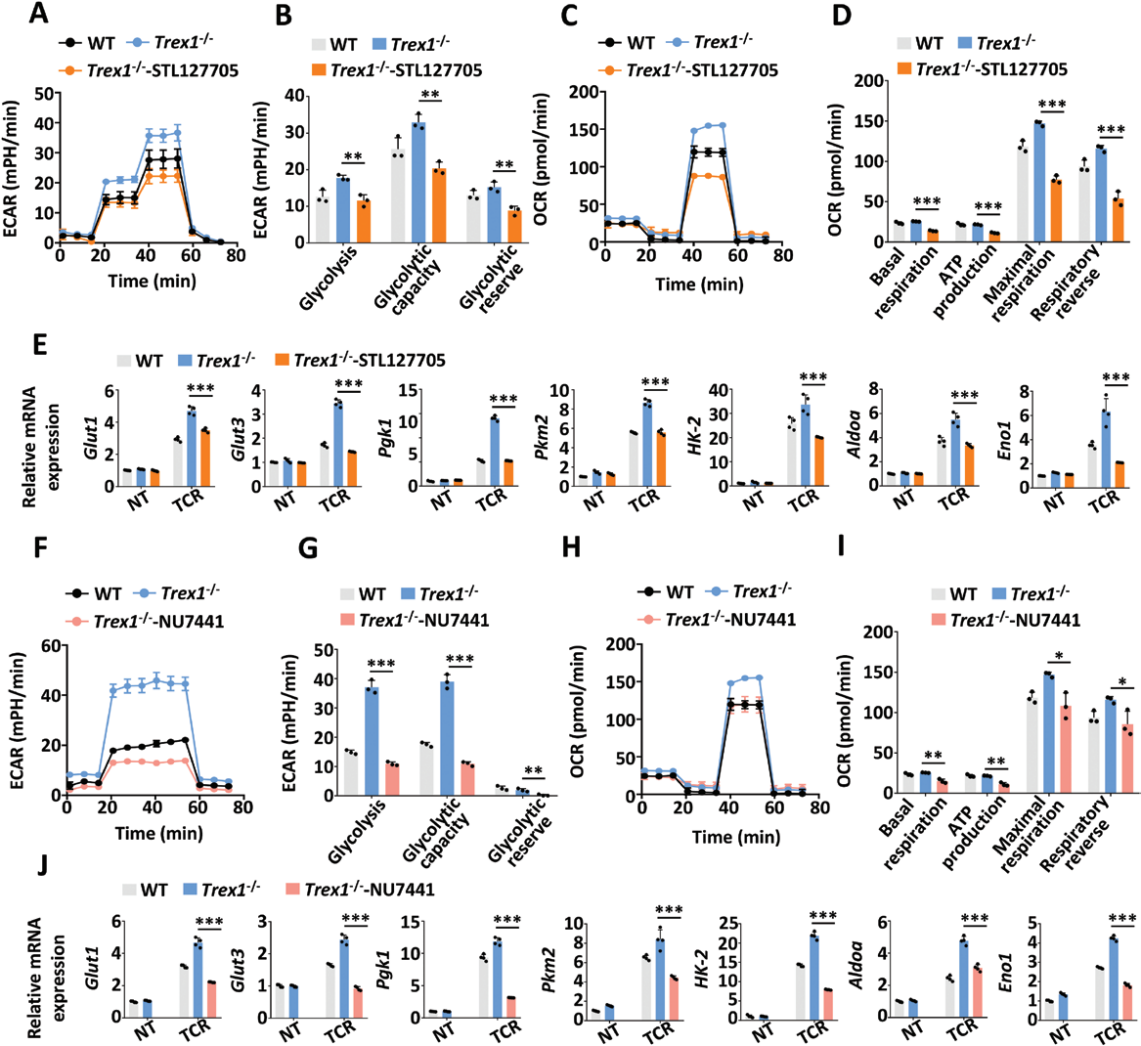
DNA-fueled glycolysis in CD4^+^ T cells could be blocked by inhibiting the KU-DNA-PKcs pathway. (A–D) ECAR and OCR analysis of WT and *Trex1*^-/-^ CD4^+^ T cells treated with STL127705 (50 μM) upon TCR stimulation for 24 h. (B, D) The statistical results are presented as a bar graph. (E) The transcriptional levels of glycolysis-related genes in WT and *Trex1*^-/-^ CD4^+^ T cells treated with STL127705 (50 μM) under TCR stimulation for 36 h. (F–I) ECAR and OCR analysis of WT and *Trex1*^-/-^ CD4^+^ T cells treated with NU7441 (1 μM) upon TCR stimulation for 24 h. (G, I) The statistical results are presented as a bar graph. (J) The transcriptional levels of glycolysis-related genes in WT and *Trex1*^-/-^ CD4^+^ T cells treated with NU7441 (1 μM) under TCR stimulation for 36 h. Statistics, two-tailed Student’s *t* test. Error bars represent SD. Differences were considered to be significant at *P* < 0.05 and are indicated by *, those at *P* < 0.01 are indicated by **, and those at *P* < 0.001 are indicated by ***.

**Figure 5.**
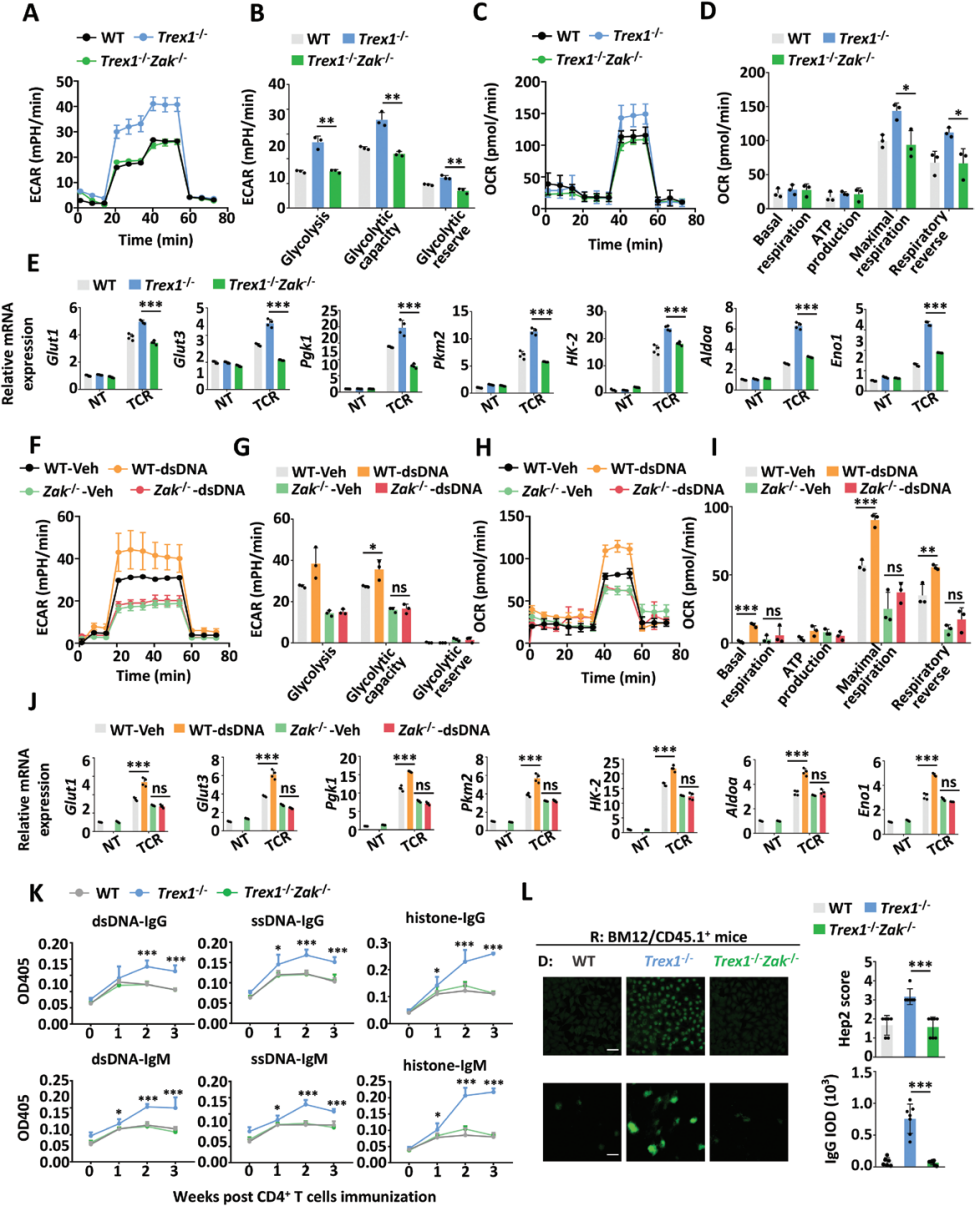
*Zak* deficiency in CD4^+^ T cells could abolish DNA-boosted glycolysis and inflammation. (A–D) ECAR and OCR analysis of WT, *Trex1*^-/-^ and *Trex1*^-/-^*Zak*^-/-^ CD4^+^ T cells upon TCR stimulation for 24 h. (B, D) The statistical results are presented as a bar graph. (E) The transcriptional levels of glycolysis-related genes in WT, *Trex1*^-/-^ and *Trex1*^-/-^*Zak*^-/-^ CD4^+^ T cells under TCR stimulation for 36 h. (F–I) ECAR and OCR analysis of WT and *Zak*^-/-^ CD4^+^ T cells transfected with dsDNA upon TCR stimulation for 24 h. (G, I) The statistical results are presented as a bar graph. (J) The transcriptional levels of glycolysis-related genes in WT and *Zak*^-/-^ CD4^+^ T cells transfected with dsDNA under TCR stimulation for 36 h. (K–L) Lupus model was induced by immunizing BM12/SJL mice (recipient, R) with WT, *Trex1*^-/-^, and *Trex1*^-/-^*Zak*^-/-^ CD4^+^ T cells (donor, D). (K) Anti-dsDNA, anti-ssDNA, and anti-histone IgG or IgM in serum from the immunized mice at the indicated time using ELISA. The level of ANA in serum (L, top, scale bar: 50 μm) and IgG deposition in the kidney (L, bottom, scale bar: 50 μm). Data are presented as immunofluorescent images (L, left) and quantification bar graphs (L, right). Statistics, two-tailed Students *t* test. Error bars represent SD. Differences were considered to be significant at *P* < 0.05 and are indicated by *, those at *P* < 0.01 are indicated by **, and those at *P* < 0.001 are indicated by ***.

Next, we examined whether the KU-ZAK pathway was involved in the regulation of glycolysis in CD4^+^ T cells. The results showed that DNA-boosted glycolysis and OXPHOS in *Trex1*^–/–^ CD4^+^ T cells upon TCR stimulation could be normalized by STL127705 (Fig. 4A–E), an inhibitor of Ku70/80-DNA interaction, and NU7441 (Fig. 4F–J), an inhibitor of DNA-PKcs, as measured by ECAR, OCR, and QPCR analysis of related genes as Glut1, Glut3, and HK-2. In order to directly verify the DNA sensor in CD4^+^ T cells, we pull down KU70 and cGAS to analyze the DNA contents. The results showed that KU70, but not cGAS could be effectively co-immunoprecipitated with cytoplasmic DNA in memory CD4^+^ T cells (Fig. S1), which is consistent with our previous work [20]. Also, *Zak* deficiency reduced glycolysis and OXPHOS in *Trex1*^–/–^CD4^+^ T cells (Fig. 5A–E). In line with the results in *Trex1*^–/–^*Zak*^–/–^ CD4^+^ T cells, transfection of dsDNA failed to intensify both glycolysis and OXPHOS in *Zak*^–/–^ CD4^+^ T cells (Fig. 5F–J). TCR stimulation could induce DNA accumulation in CD4^+^ T cells as our previous study revealed [20], which can explain why *Zak*^–/–^ CD4^+^ T cells also showed lower glycolysis and OXPHOS without transfection of dsDNA or deficiency of *Trex1* under TCR activation.

To verify the role of ZAK in regulating DNA-induced glycolysis *in vivo*, we adoptively transferred WT, *Trex1*^–/–^and *Trex1*^–/–^*Zak*^–/–^CD4^+^ T cells into BM12 mice. The results showed that *Zak* deficiency could reverse the SLE phenotype induced by immunizing with *Trex1*^-/-^ CD4^+^ T cells, as displayed by decreased percentages of Tfh, GC B, and plasma cells in the spleen (Fig. S2A and S2B), the reduced production of anti-dsDNA/ssDNA/histone IgG and IgM in the serum (Fig. 5K), the less IgG deposition in the kidney, and the lower levels of ANA in the serum (Fig. 5L).

**Figure 6.**
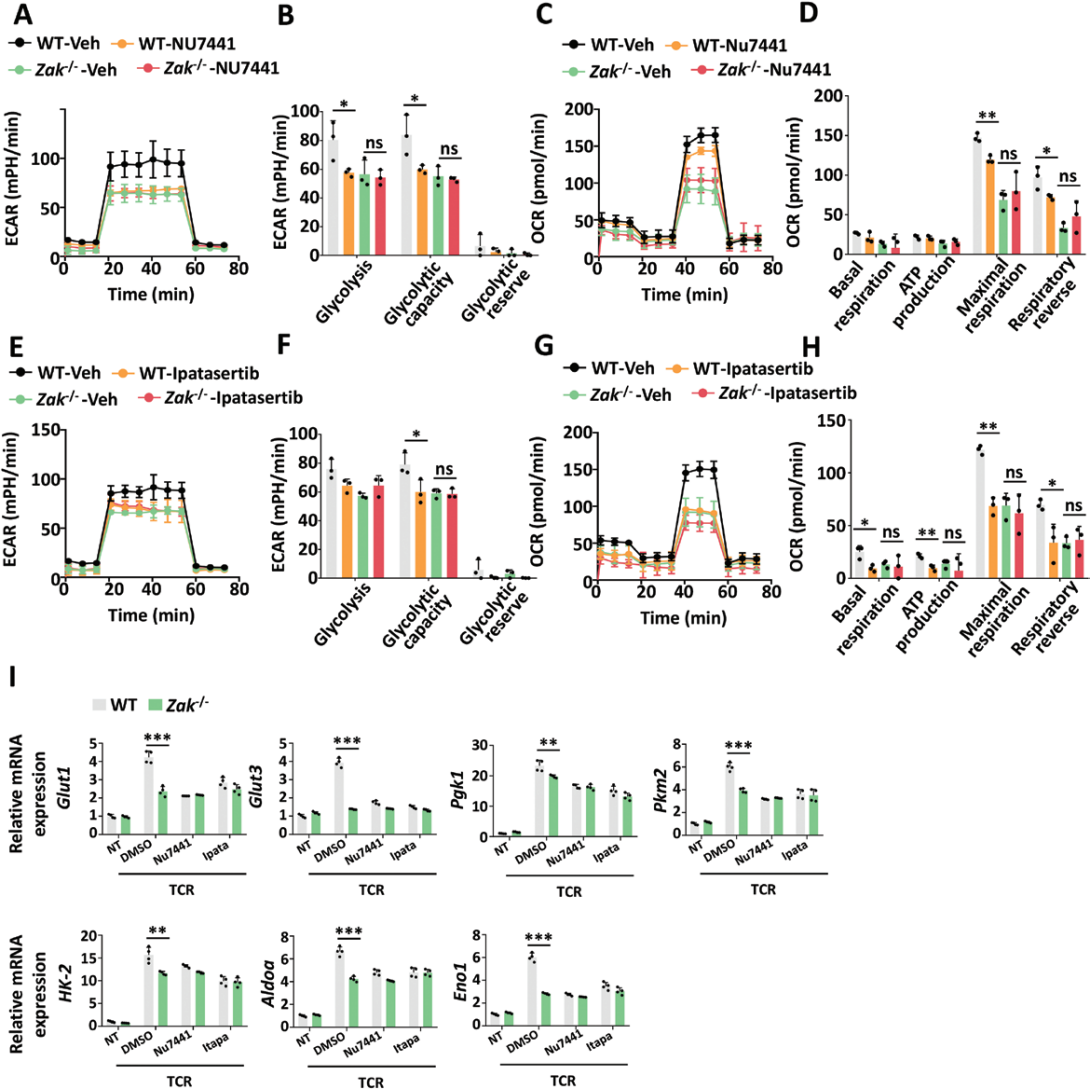
DNA-PKcs-ZAK-AKT pathway mediates DNA sensing involved in glycolysis in CD4^+^ T cells. (A–D) ECAR and OCR analysis of WT and *Zak*^-/-^ CD4^+^ T cells treated with NU7441 (1 μM) under TCR activation for 24 h. (B, D) The statistical results are presented as a bar graph. (E–H) ECAR and OCR analysis of WT and *Zak*^-/-^ CD4^+^ T cells treated with Ipatasertib (2 μM) under TCR activation for 24 h. (F, H) The statistical results are presented as a bar graph. (I) The transcriptional levels of glycolysis-related genes in WT and *Zak*^-/-^ CD4^+^ T cells treated with NU7441 (1 μM) or Ipatasertib (2 μM) *ex vivo* and under TCR stimulation (α-CD3/28, 1 μg/mL, TCR) for 36 h. Statistics, two-tailed Students *t* test. Error bars represent SD. Differences were considered to be significant at *P* < 0.05 and are indicated by *, those at *P* < 0.01 are indicated by **, and those at *P* < 0.001 are indicated by ***.

In order to certify the role of ZAK in the DNA sensing pathway, we found that NU7741 reduced glycolysis and OXPHOS in WT CD4^+^ T cells, using analysis of ECAR, OCR, and glycolysis-related gene expression, but had little impact on *Zak*^–/–^ CD4^+^ T cells (Fig. 6A–D, I). A recent work showed that AKT is the downstream of the KU-ZAK pathway and engagement of AKT enables cellular metabolism reprogramming to meet proliferation and effector functions [36–38]. Consistent with those findings, our results showed that inhibition of AKT using a pan-AKT inhibitor Ipatasertib, also lowered glycolysis in WT CD4^+^ T cells but did not impair *Zak*^–/–^CD4^+^ T cells (Fig. 6E–H, I). Collectively, we confirmed that cytoplasmic DNA was sensed by the KU complex and then potentiated metabolism through the initiating DNA-PKcs response, followed by activation of ZAK and AKT. These results revealed that blocking DNA sensing using inhibition of the KU/DNA-PKcs/ZAK/AKT pathway could reverse SLE diseases mainly through preventing metabolism reprogramming in activated CD4^+^ T cells.

### Inhibiting DNA sensing pathway is potential for SLE therapy

**Figure 7.**
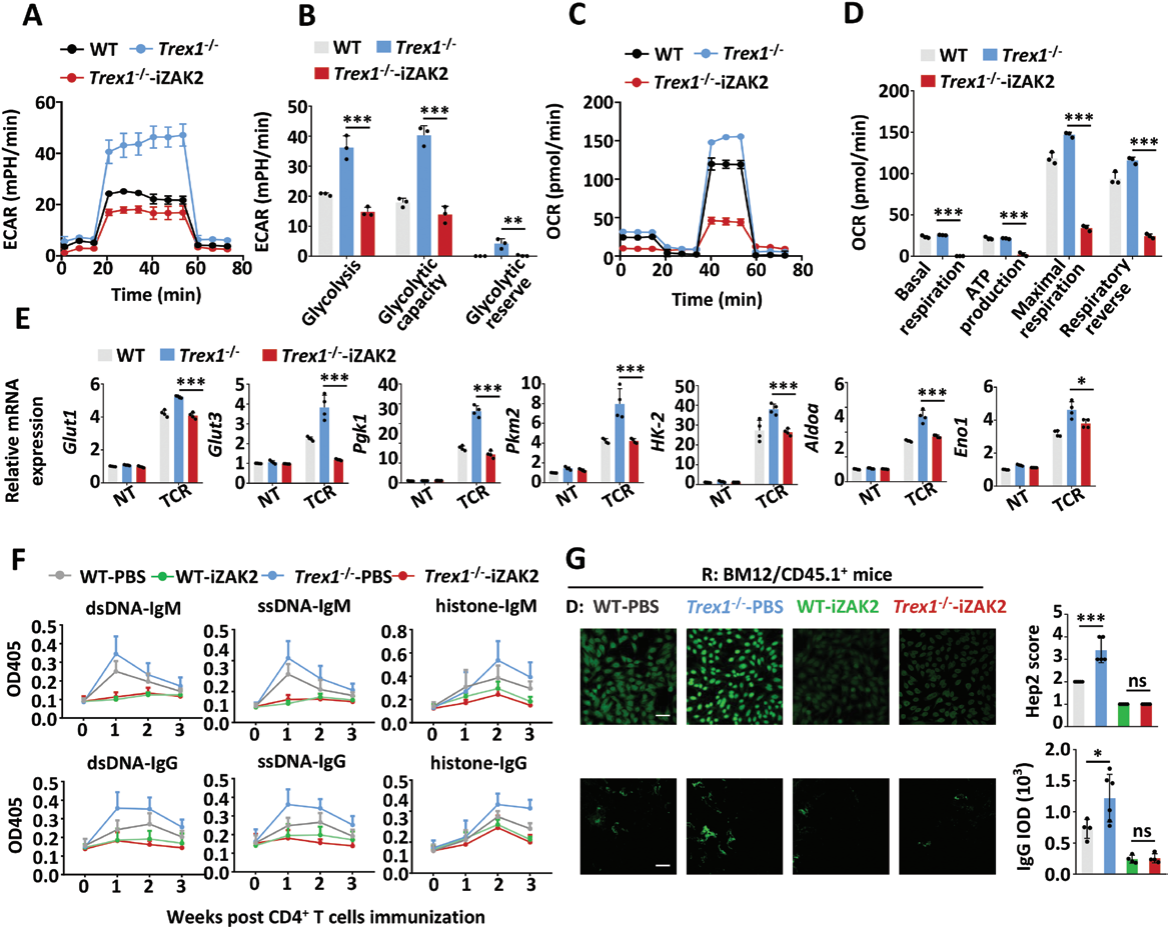
Targeting ZAK could ameliorate accumulated-DNA-induced inflammation. (A–D) ECAR and OCR analysis of WT and *Trex1*^–/–^ CD4^+^ T cells treated with iZAK2 (1 μM) under TCR activation for 24 h. (B, D) The statistical results are presented as a bar graph. (E) The transcriptional levels of glycolysis-related genes in WT and *Trex1*^–/–^ CD4^+^ T cells treated with iZAK2 (1 μM) *ex vivo* and under TCR stimulation (α-CD3/28, 1 μg/mL, TCR) for 36 h. (F–G) BM12/SJL mice (recipient, R) were immunized with WT and *Trex1*^–/–^ CD4^+^ T cells (donor, D), and then intraperitoneally injected once every two days with iZAK2 (50 mg/kg) or vehicle as control for 3 weeks. (F) The anti-dsDNA, anti-ssDNA, and anti-histone IgG or IgM in serum were examined by ELISA. ANA in serum (G top, scale bar: 50 μm) and the kidney IgG deposition (G bottom, scale bar: 50 μm) were visualized using immunofluorescence. Data are presented as Immunofluorescent images (G left) and quantification bar graphs (G right). Statistics, two-tailed Students *t* test. Error bars represent SD. Differences were considered to be significant at *P* < 0.05 and are indicated by *, those at *P* < 0.01 are indicated by **, and those at *P* < 0.001 are indicated by ***.

Our recent work has screened an inhibitor targeting ZAK, named iZAK2, which has been shown could abolish the DNA-boosted enhancement of AKT activation and cell proliferation in CD4^+^ T cells, further to attenuate EAE-related autoimmune symptoms in aged mice [20]. Here, we tested the effect of iZAK2 in the glycolysis of CD4^+^ T cells and the pathological symptoms of SLE. *In vitro*, ECAR, OCR, and QPCR analysis confirmed the reduced glycolysis and OXPHOS in *Trex1*^–/–^ CD4^+^ T cells treated with iZAK2 (Fig. 7A–E). Also, we excluded the toxicity of iZAK2 using apoptosis analysis (Fig. S3A). *In vivo*, treatment with iZAK2 attenuated the SLE symptoms in a lupus-like inflammation model induced in BM12 mice transferred with *Trex1*^–/–^CD4^+^ T cells, as measured by anti-dsDNA/ssDNA/histone IgG or IgM and ANA in the serum (Fig. 7F), the frequencies of Tfh, GC B and plasma cells in the spleen ( Fig. S3B and S3C), and the IgG deposition in the kidney (Fig. 7G). Therefore, these results support that targeting the DNA sensing pathway using iZAK2 may be a potential therapeutic strategy for autoimmune diseases such as EAE and SLE.

## Discussion

A mass of studies performed in mutant mice deficient in self-nucleic-acid clearance has revealed a causal link between the overactivation of the nucleic acid-sensing system in innate immune cells and autoimmune diseases. And multiple individual pathways can coverage on a similar clinical presentation. For example, the deficiencies of Trex1, all components of RNase, and SAMHD1, leading to AGS, are known to be upstream of the cGAS-STING pathway [5, 9, 39–41]. The mutations of DNase Ⅰ have been identified in mice and human with SLE, which are involved in MyD88-dependent TLR signaling [42, 43]. The Adar-deficiency mice showed a massive type Ⅰ IFN signaling, which is driven by the MDA5-MAVS pathway, leading to embryonic lethality [44, 45]. These results underscore the importance of identifying pathway-specific dysregulation in treating autoimmune diseases such as AGS and SLE. Therefore, the inhibition of cGAS-STING is a promising therapeutic target for autoimmune diseases, but is limited to the diseases that are cGAS-dependent.

Here, our work showed that an overactivated DNA sensing pathway, clarified as the KU-ZAK system, in *Trex1*-deficient CD4^+^ T cells successfully induced an autoimmune disease with the symptoms similar to SLE, suggesting the DNA sensing system in adaptive immune cells is also critical for the development of SLE. Moreover, an inhibitor of ZAK, iZAK2 could suppress pathogenic characteristics of lupus-like inflammation, and our previous work has shown that iZAK2 could also ameliorate autoimmune inflammation in aged mice [20], both of which support the potential therapeutic benefits of iZAK2. Considering that the engagement of innate immune precedes and ignites the adaptive immune response, and activated T helper cells are the major mediators in autoimmune diseases [1], the application range of iZAK2 may be wider than cGAS-STING inhibitors.

cGAS performs a universal sensing mechanism to recognize a wide range of dsDNA in the cytosol including those leaking out from the nucleus and mitochondria, or acquired from the extracellular microenvironment, and pathogenic ones. The process is in a sequence-independent manner and the longer dsDNA binds more stable with cGAS [46]. DNA-PK complex is composed of a DNA-PK catalytic subunit (DNA-PKcs) and KU heterodimer consisting of KU70 and KU80, the main role of which is in DNA repair through non-homologous end joining (NHEJ). DNA-PK binds to and is activated by the end of dsDNA, thus acting as the sensor for the end of dsDNA. As cGAS, KU binds to the end of dsDNA without any particular preference in the nucleotide sequence and then recruits DNA-PKcs via the C-terminal region of KU80. NHEJ is also implicated in the process of V(D)J recombination in the immune system, ensuring the enormous diversity of immunoglobulins and T cell receptors [47, 48]. The different cellular functions and DNA recognition models of the two systems may account for the different roles of the two in CD4^+^ T cells. In consistent with the hypothesis, our previous work showed lower expression of cGAS than DNA-PK complex in T cells.

The activated cGAS drives the synthesis of cGAMP and engages STING to initiate the induction of type Ⅰ IFN and extensive array of ISGs, which presents canonical cGAS-STING signaling accompanied by activation of NF-κB signaling to encode pro-inflammatory cytokines [46]. Considering that inflammation is a high energy-expenditure process and reprograms the metabolism in immune cells, it is reasonable to suppose that the activation of the cGAS/STING system link to cellular metabolism. Recent studies have shown that *Trex1* KO BMDM (bone marrow derived macrophage), with elevated cGAS-STING signaling, reduced mitochondrial respiration but increased glycolysis [49]. DNA-PKcs shows the structural similarity to phosphatidylinositol 3-kinase (PI3K) and assembles the phosphatidyl inositol 3-kinase-like protein kinases family [48]. PI3Ks mediate the activation of AKT protein and mTOR, the important pathway controlling multiple cellular processes. DNA-PKcs is shown to activate AKT in response to DNA damage and promote cell survival [50, 51]. Also, our previous work showed activated DNA-PKcs phosphorylates ZAK and further activates AKT to promote T cell proliferation under TCR activation or in aged T cells with dsDNA accumulation [20]. According to the studies, DNA sensing signaling engages in regulating multiple cellular processes including metabolic reprogramming.

Cell-intrinsic metabolic programs, including energy production, nutrient utilization, and metabolite biosynthesis, are considered important risks for inflammatory [52]. In rheumatoid arthritis, inhibiting fatty-acid oxidation or restoring oxidant signaling by using pro-oxidants menadione and buthionine sulfoximine could suppress synovial inflammation [53]. Inhibiting PFKFB3 with 3-PO, a rate-limiting enzyme in glycolysis, prevents the T cell-mediated delayed hypersensitivity [54, 55]. 2-DG treatment, inhibiting glycolysis, could suppress the development of EAE and SLE [28, 29, 56]. Thus, targeting metabolism or the signaling pathways engaged in metabolic adaption is a potential strategy for treating autoimmune disease.

**Figure 8.**
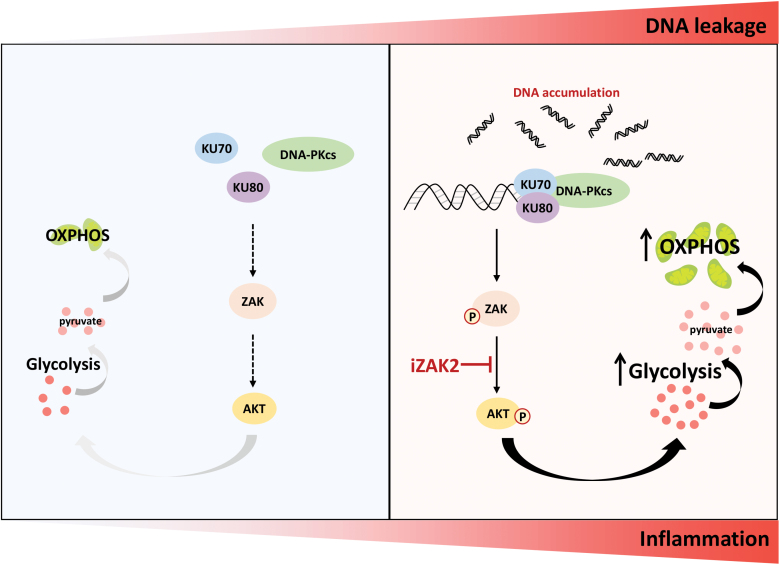
The model of DNA-boosted CD4^+^ T cell metabolism. DNA leakage occurs with inflammation, leading to DNA accumulation in CD4^+^ T cells. Accumulated DNA in CD4^+^ T cells is sensed by KU70/KU80-DNA-PKcs complex and then activates ZAK, further to activate AKT and enhance glycolysis and OXPHOS, which fuels the pro-inflammatory activity of CD4^+^ T cells.

Our data emphasized the contribution of accumulated DNA to metabolism reprogramming in CD4^+^ T cells. DNA accumulation in *Trex1*^-/-^ CD4^+^ T cells or induced by transfecting dsDNA in WT CD4^+^ T cells, exhibited higher glycolysis and OXPHOS upon TCR activation. Mechanistically, we found that accumulated DNA was sensed by the KU complex, activated DNA-PKcs and ZAK, then triggered the activation of AKT and further facilitated metabolism (Fig. 8). Consistently, *in vivo*, administration of either iZAK2 or 2-DG could attenuate pathogenic characteristics in a lupus-like inflammation model induced with *Trex1*^-/-^ CD4^+^ T cells.

Considering that TCR activation promotes DNA accumulation in the cytoplasm in CD4^+^ T cells as reported before [20], and in our results, CD4^+^ T cells with *Zak* deficiency also reduced glycolysis and OXPHOS under TCR stimulation. We proposed that the engagement of DNA-sensing in metabolism reprogramming was coupled with the initiation of TCR signaling. According to our data, we suggest that there may be a positive feedback loop in CD4^+^ T cells regulating autoimmune disease. *Trex1* deficiency is linked to spontaneous inflammation in multiple organs [3, 5, 9, 31, 32], in which CD4^+^ T cells are exposed to a pro-inflammatory environment with initiating effector function and metabolism reprogramming. Then activated CD4^+^ T cells augment glycolysis due to the accumulated DNA, and that further leads to sever pathogenic symptoms.

In summary, our data clarified that DNA accumulation in CD4^+^ T cells triggered glycolysis, and then caused autoimmune diseases, which may be a new explanation for harboring DNA metabolism leading to autoimmune diseases. Here, we also identified that the ZAK inhibitor, iZAK2 could ameliorate autoinflammatory by reducing glycolysis. Further research and development for this inhibitor to block DNA sensing pathway in the adaptive immune system may be beneficial for autoimmune disease therapy.

## Research limitations

Overall, our study clarified a novel mechanism that DNA accumulation in CD4^+^ T cell potentiates glycolysis and OXPHOS, which also explains the nosogenesis of DNA accumulation leading to autoimmunity diseases. However, whether or not there is a structure preference for KU-mediated DNA sensing remains to be explored. We also failed to clarify the source of cytosolic DNA, from leaking out of nuclear and mitochondria or acquired from the extracellular matrix. Therefore, it is uncertain whether the mechanism occurs disease limitations due to the specific biological characteristics of DNA sensed by KU. And the correlation among DNA contents, T cell metabolism activity, and severity index of SLE remains to be explored. In addition, despite iZAK2 showing therapeutic potential, more research for feasibility should be taken.

## Methods

### Mice

*Zak* flox mice were generated from Shanghai Research Center for Model Organisms and then were backcrossed with C57BL/6 mice at least for six generations. The C57BL/6 background *Zak* flox mice were crossed with CD4-Cre mice to produce T cell-conditional Zak-knockout mice. *Trex1*^–/–^ mice were generated by microinjection of four independent sgRNAs together with Cas9 nuclease mRNA into *in vitro* fertilized oocytes of C57BL/6 mice as described in our previous study. Cgas^–/–^ and Sting^–/–^ mice were as previously described [20]. BM12 mice were provided by Dr. N. Shen (Shanghai Institutes for Biological Sciences, Chinese Academy of Sciences). SJL mice were purchased from Shanghai Model Organisms Center. In some experiments, BM12 transgenic mice were crossed with SJL transgenic to generate BM12/SJL mice. All mice were maintained in a specific pathogen-free facility.

### T cell isolation and activation

Murine CD4^+^ T cells were isolated from the spleen and peripheral lymph nodes of mice with Mouse CD4^+^ T cell isolation kit (Biolegend). For metabolic measurements, isolated CD4^+^ T cells were subjected to anti-CD3 (1 μg/mL) and anti-CD28 (1 μg/mL) stimulation with the indicated treatment or not for 24 h. For qRT-PCR, CD4^+^ T cells were stimulated with plate-bound anti-CD3 (1 μg/mL) and anti-CD28 (1 μg/mL) with indicated treatment for the indicated time. For inhibitor treatment, CD4^+^ T cells were treated with Ipatasertib (2 μM), Nu7441 (1 μM), iZAK2 (1 μM), and STL12205 (50 μM). For the transfection of dsDNA, CD4^+^ T cells were seeded in 96-well-plate and transfected with calf thymus DNA using Lipofectamine 2000 according to the manufacturers instruction. For DNase Ⅰ treatment, 10 mg/mL DNase I was supplemented in the culture medium.

### Induction of lupus-like disease model

The induction of the lupus-like disease model was as previously described [20, 35]. In brief, 7.5 million purified CD4^+^ T cells from WT, *Trex1*^–/–^ and *Trex1*^–/–^*Zak*^–/–^ mice were intravenously injected into BM12/SJL mice. For treatment of inhibitors, mice were intraperitoneally (i.p.) injected once a day with 2-DG (200 mg/kg) or once every 2 days with iZAK2 (50 mg/kg) and the same amount of vehicle (5% DMSO + 95% saline) for 3 weeks, which was initiated at the first day of model induction.

### Antibody measurement

The sera were collected at first, second, and third week of the lupus model and measured anti-dsDNA, anti-ssDNA, and anti-histone antibodies by enzyme-linked immunosorbent assay (ELISA). ANAs were detected using Hep-2 ANA kits (INOVA Diagnostic) following the manufacturers instructions.

### Renal pathology

Formalin-fixed frozen mouse kidney sections were stained with Alexa Fluor 488-conjugated goat anti-mouse IgG. Antibody staining was detected using an LSM880 confocal microscopy and the fluorescence intensity was determined using Image J.

### Metabolic measurements

Extracellular acidification rates (ECARs) and oxygen consumption rates (OCRs) were measured using an XF^e^24 or an XF^e^96 Extracellular Flux Analyzer (Seahorse Bioscience). For ECAR assay, cells were resuspended in basic media supplemented with 2 mM L-glutamine (Gibco) and analyzed under basal conditions followed by treatment with under-mentioned agents: glucose (10 mM); the ATP synthase inhibitor oligomycin (2 μM); and glucose analog 2-DG (100 mM). For the OCR assay, cells were resuspended in basic media supplemented with 10 mM glucose, 2 mM l-glutamine (Gibco), and 1 mM sodium pyruvate (Gibco) and analyzed under basal conditions following treatment with agents: the ATP synthase inhibitor oligomycin (2 μM); the protonophore carbonyl cyanide-4-(trifluoromethoxy) phenylhydrazone (FCCP) (1 μM) to uncouple mitochondria; the mitochondrial complex I inhibitor rotenone (500 nM); and the mitochondrial complex III inhibitor antimycin A (500 nM).

### Real-time quantitative RT-PCR (qRT-PCR)

Total RNA was extracted using TRIzol reagent and subjected to cDNA synthesis using PrimeScript RT Reagent Kit (Takara). qRT-PCR was performed on QuantStudio 7 Flex Real-Time PCR System (Applied Biosystems) with SYBR Green Master Mix (Novoprotein). The expression of individual genes was calculated by a standard curve method and normalized to the expression of Actb. The primers used for PCR analysis were presented in Table S1.

### Quantification of cytoplasmic DNA and co-immunoprecipitated (coIP) DNA

Cytoplasmic DNA and co-immunoprecipitated DNA quantification were performed as previously described [20]. In brief, CD4^+^ T cells were divided into two equal aliquots. One aliquot was resuspended in 300 mL of lysis buffer (10 mM Tris–HCl pH 8.0, 100 mM NaCl, 25 mM EDTA, and 0.5% SDS), which acted as normalization controls for total cellular DNA. The second equal aliquots were resuspended in 300 mL of permeabilization buffer (50 mM HEPES pH 7.4, 150 mM NaCl, 2 mM EDTA, and 50 mg/mL digitonin) following plasma membrane permeabilization with 10-min incubation on ice. DNA from whole-cell lysates or cytoplasmic extract was obtained with phenol-chloroform and precipitated with alcohol, and then quantified by NanoDrop2000 (Thermo). Cytoplasmic DNA values were normalized to total cellular DNA abundance for whole-cell extracts to account for the variations of cell number among samples. For extraction of co-immunoprecipitated DNA, the cytoplasmic extract was incubated with anti-Ku70 or anti-cGAS antibody, respectively. After incubation at 4℃ for 4 h on a rotator, protein-A/G magnetic beads were added into tubes to pull down the target protein. Finally, samples were washed extensively and then subjected to DNA extraction using a TIANamp Micro DNA Kit. Quantification of coIP DNA was performed by real-time PCR. The coIP cytoplasmic DNA levels were normalized with total cellular DNA. Real-time PCR was performed to detect DNA levels by using specific primers targeted to L1, Tert, and Dloop1 genes. The primers used for PCR analysis were presented in Table S1.

### Flow cytometry

For analysis of surface markers, cells were stained in PBS on ice for 30 min. For cell apoptosis measurement, cells were stained in 1×binding buffer (BD Biosciences) using annexin Ⅴ-PE (1:100) as manufacturers instructions. Data collection was performed on a Beckman Gallios cytometer and analyzed using FlowJo software.

### Research ethics

All animal experiments were complied with all relevant ethical regulations for animal testing and research and were in accordance with protocols approved by the institutional Biomedical Research Ethics Committee, Shanghai Institute of Nutrition and Health, Chinese Academy of Sciences.

### Statistical analysis

The data are shown as mean ± SD, and unless otherwise indicated, all the presented data were the representative results of at least three independent repeats. Statistical analysis was performed by using GraphPad Prism 8 (Graph-Pad Software), and the statistics were analyzed by a two-tailed Students *t* test as indicated. Differences were considered to be significant at *P* < 0.05 and are indicated by *, those at *P* < 0.01 are indicated by **, and those at *P* < 0.001 are indicated by ***.

## Data availability

All data that supported the findings of this study are available within the article and its supplementary materials.

## Supplementary Material

lnad021_suppl_Supplementary_Material

## References

[1] Theofilopoulos AN, Kono DH, Baccala R. The multiple pathways to autoimmunity. Nat Immunol 2017;18:716–24. 28632714 10.1038/ni.3731 PMC5791156

[2] Crowl JT, Gray EE, Pestal K, et al. Intracellular nucleic acid detection in autoimmunity. Annu Rev Immunol 2017;35:313–36. 28142323 10.1146/annurev-immunol-051116-052331 PMC6435037

[3] Lehtinen DA, Harvey S, Mulcahy MJ, et al. The TREX1 double-stranded DNA degradation activity is defective in dominant mutations associated with autoimmune disease. J Biol Chem 2008;283:31649–56. 18805785 10.1074/jbc.M806155200 PMC2581595

[4] Crow YJ, Chase DS, Lowenstein Schmidt J, et al. Characterization of human disease phenotypes associated with mutations in TREX1, RNASEH2A, RNASEH2B, RNASEH2C, SAMHD1, ADAR, and IFIH1. Am J Med Genet A 2015;167A:296–312. 25604658 10.1002/ajmg.a.36887 PMC4382202

[5] Lee-Kirsch MA, Gong M, Chowdhury D, et al. Mutations in the gene encoding the 3'-5' DNA exonuclease TREX1 are associated with systemic lupus erythematosus. Nat Genet 2007;39:1065–7. 17660818 10.1038/ng2091

[6] Li Y, Shen Y, Jin K, et al. The DNA repair nuclease MRE11A functions as a mitochondrial protector and prevents T cell pyroptosis and tissue inflammation. Cell Metab 2019;30:477–492.e6. 31327667 10.1016/j.cmet.2019.06.016 PMC7093039

[7] Melki I, Allaeys I, Tessandier N, et al. Platelets release mitochondrial antigens in systemic lupus erythematosus. Sci Transl Med 2021;13:eaav5928. 33597264 10.1126/scitranslmed.aav5928

[8] Ablasser A, Chen ZJ. cGAS in action: expanding roles in immunity and inflammation. Science 2019;363:eaat8657. 30846571 10.1126/science.aat8657

[9] Gao D, Li T, Li X-D, et al. Activation of cyclic GMP-AMP synthase by self-DNA causes autoimmune diseases. Proc Natl Acad Sci U S A 2015;112:E5699–5705. 26371324 10.1073/pnas.1516465112 PMC4620884

[10] Roers A, Hiller B, Hornung V. Recognition of endogenous nucleic acids by the innate immune system. Immunity 2016;44:739–54. 27096317 10.1016/j.immuni.2016.04.002

[11] Dai J, Huang Y-J, He X, et al. Acetylation blocks cGAS activity and inhibits self-DNA-induced autoimmunity. Cell 2019;176:1447–1460.e14. 30799039 10.1016/j.cell.2019.01.016 PMC8274936

[12] Perrino FW, Harvey S, Shaban NM, et al. RNaseH2 mutants that cause Aicardi-Goutieres syndrome are active nucleases. J Mol Med (Berl) 2009;87:25–30. 19034401 10.1007/s00109-008-0422-3 PMC2852111

[13] Reijns MA, Rabe B, Rigby RE, et al. Enzymatic removal of ribonucleotides from DNA is essential for mammalian genome integrity and development. Cell 2012;149:1008–22. 22579044 10.1016/j.cell.2012.04.011 PMC3383994

[14] Tan EM, Kunkel HG. Characteristics of a soluble nuclear antigen precipitating with sera of patients with systemic lupus erythematosus. J Immunol 1966;96:464–71. 5932578

[15] Duvvuri B, Lood C. Cell-free DNA as a biomarker in autoimmune rheumatic diseases. Front Immunol 2019;10:502. 30941136 10.3389/fimmu.2019.00502 PMC6433826

[16] Courtney PA, Crockard AD, Williamson K, et al. Increased apoptotic peripheral blood neutrophils in systemic lupus erythematosus: relations with disease activity, antibodies to double stranded DNA, and neutropenia. Ann Rheum Dis 1999;58:309–14. 10225817 10.1136/ard.58.5.309 PMC1752888

[17] Lood C, Blanco LP, Purmalek MM, et al. Neutrophil extracellular traps enriched in oxidized mitochondrial DNA are interferogenic and contribute to lupus-like disease. Nat Med 2016;22:146–53. 26779811 10.1038/nm.4027 PMC4742415

[18] Mobarrez F, Vikerfors A, Gustafsson JT, et al. Microparticles in the blood of patients with systemic lupus erythematosus (SLE): phenotypic characterization and clinical associations. Sci Rep 2016;6:36025. 27777414 10.1038/srep36025 PMC5078765

[19] Leadbetter EA, Rifkin IR, Hohlbaum AM, et al. Chromatin-IgG complexes activate B cells by dual engagement of IgM and Toll-like receptors. Nature 2002;416:603–7. 11948342 10.1038/416603a

[20] Wang Y, Fu Z, Li X, et al. Cytoplasmic DNA sensing by KU complex in aged CD4(+) T cell potentiates T cell activation and aging-related autoimmune inflammation. Immunity 2021;54:632–647.e9. 33667382 10.1016/j.immuni.2021.02.003

[21] O’Neill LA, Kishton RJ, Rathmell J. A guide to immunometabolism for immunologists. Nat Rev Immunol 2016;16:553–65. 27396447 10.1038/nri.2016.70 PMC5001910

[22] Geltink RIK, Kyle RL, Pearce EL. Unraveling the complex interplay between T cell metabolism and function. Annu Rev Immunol 2018;36:461–88. 29677474 10.1146/annurev-immunol-042617-053019 PMC6323527

[23] Morel L. Immunometabolism in systemic lupus erythematosus. Nat Rev Rheumatol 2017;13:280–90. 28360423 10.1038/nrrheum.2017.43

[24] Weyand CM, Goronzy JJ. Immunometabolism in early and late stages of rheumatoid arthritis. Nat Rev Rheumatol 2017;13:291–301. 28360422 10.1038/nrrheum.2017.49 PMC6820517

[25] Kolev M, Dimeloe S, Le Friec G, et al. Complement regulates nutrient influx and metabolic reprogramming during Th1 cell responses. Immunity 2015;42:1033–47. 26084023 10.1016/j.immuni.2015.05.024 PMC4518498

[26] Macintyre AN, Gerriets VA, Nichols AG, et al. The glucose transporter Glut1 is selectively essential for CD4 T cell activation and effector function. Cell Metab 2014;20:61–72. 24930970 10.1016/j.cmet.2014.05.004 PMC4079750

[27] Hochrein SM, Wu H, Eckstein M, et al. The glucose transporter GLUT3 controls T helper 17 cell responses through glycolytic-epigenetic reprogramming. Cell Metab 2022;34:516–532.e11. 35316657 10.1016/j.cmet.2022.02.015 PMC9019065

[28] Yin Y et al. Normalization of CD4+ T cell metabolism reverses lupus. Sci Transl Med 2015;7:274ra218. 10.1126/scitranslmed.aaa0835 PMC5292723 25673763

[29] Yin Y, Choi S-C, Xu Z, et al. Glucose oxidation is critical for CD4+ T cell activation in a mouse model of systemic lupus erythematosus. J Immunol 2016;196:80–90. 26608911 10.4049/jimmunol.1501537 PMC4684991

[30] Li W, Qu G, Choi S-C, et al. Targeting T cell activation and lupus autoimmune phenotypes by inhibiting glucose transporters. Front Immunol 2019;10:833. 31057554 10.3389/fimmu.2019.00833 PMC6478810

[31] Yang YG, Lindahl T, Barnes DE. Trex1 exonuclease degrades ssDNA to prevent chronic checkpoint activation and autoimmune disease. Cell 2007;131:873–86. 18045533 10.1016/j.cell.2007.10.017

[32] Grieves JL, Fye JM, Harvey S, et al. Exonuclease TREX1 degrades double-stranded DNA to prevent spontaneous lupus-like inflammatory disease. Proc Natl Acad Sci U S A 2015;112:5117–22. 25848017 10.1073/pnas.1423804112 PMC4413332

[33] Sharabi A, Tsokos GC. T cell metabolism: new insights in systemic lupus erythematosus pathogenesis and therapy. Nat Rev Rheumatol 2020;16:100–12. 31949287 10.1038/s41584-019-0356-x

[34] Klarquist J, Janssen EM. The bm12 inducible model of systemic lupus erythematosus (SLE) in C57BL/6 mice. J Vis Exp 2015;105:e53319. 10.3791/53319 PMC4692688 26554458

[35] Liu J, Huang X, Hao S, et al. Peli1 negatively regulates noncanonical NF-kappaB signaling to restrain systemic lupus erythematosus. Nat Commun 2018;9:1136. 29555915 10.1038/s41467-018-03530-3 PMC5859150

[36] Wofford JA, Wieman HL, Jacobs SR, et al. IL-7 promotes Glut1 trafficking and glucose uptake via STAT5-mediated activation of Akt to support T-cell survival. Blood 2008;111:2101–11. 18042802 10.1182/blood-2007-06-096297 PMC2234050

[37] Crompton JG, Sukumar M, Restifo NP. Targeting Akt in cell transfer immunotherapy for cancer. Oncoimmunology 2016;5:e1014776. 27757294 10.1080/2162402X.2015.1014776 PMC5048755

[38] Jacobs SR, Herman CE, Maciver NJ, et al. Glucose uptake is limiting in T cell activation and requires CD28-mediated Akt-dependent and independent pathways. J Immunol 2008;180:4476–86. 18354169 10.4049/jimmunol.180.7.4476 PMC2593791

[39] Crow YJ, Leitch A, Hayward BE, et al. Mutations in genes encoding ribonuclease H2 subunits cause Aicardi-Goutieres syndrome and mimic congenital viral brain infection. Nat Genet 2006;38:910–6. 16845400 10.1038/ng1842

[40] Maelfait J, Bridgeman A, Benlahrech A, et al. Restriction by SAMHD1 limits cGAS/STING-dependent innate and adaptive immune responses to HIV-1. Cell Rep 2016;16:1492–501. 27477283 10.1016/j.celrep.2016.07.002 PMC4978700

[41] Gray EE, Treuting PM, Woodward JJ, et al. Cutting edge: cGAS is required for lethal autoimmune disease in the trex1-deficient mouse model of Aicardi-Goutieres syndrome. J Immunol 2015;195:1939–43. 26223655 10.4049/jimmunol.1500969 PMC4546858

[42] Sisirak V, Sally B, D'Agati V, et al. Digestion of chromatin in apoptotic cell microparticles prevents autoimmunity. Cell 2016;166:88–101. 27293190 10.1016/j.cell.2016.05.034 PMC5030815

[43] Marshak-Rothstein A, Rifkin IR. Immunologically active autoantigens: the role of toll-like receptors in the development of chronic inflammatory disease. Annu Rev Immunol 2007;25:419–41. 17378763 10.1146/annurev.immunol.22.012703.104514

[44] Liddicoat BJ, Piskol R, Chalk AM, et al. RNA editing by ADAR1 prevents MDA5 sensing of endogenous dsRNA as nonself. Science 2015;349:1115–20. 26275108 10.1126/science.aac7049 PMC5444807

[45] Hartner JC, Walkley CR, Lu J, et al. ADAR1 is essential for the maintenance of hematopoiesis and suppression of interferon signaling. Nat Immunol 2009;10:109–15. 19060901 10.1038/ni.1680 PMC2701568

[46] Chen C, Xu P. Cellular functions of cGAS-STING signaling. Trends Cell Biol 2022. 10.1016/j.tcb.2022.11.001 36437149

[47] Matsumoto Y. Development and evolution of DNA-dependent protein kinase inhibitors toward cancer therapy. Int J Mol Sci 2022;23:4264. 35457081 10.3390/ijms23084264 PMC9032228

[48] Chen S, Lees-Miller JP, He Y, et al. Structural insights into the role of DNA-PK as a master regulator in NHEJ. Genome Instab Dis 2021;2:195–210. 34723130 10.1007/s42764-021-00047-w PMC8549938

[49] Hasan M, Gonugunta VK, Dobbs N, et al. Chronic innate immune activation of TBK1 suppresses mTORC1 activity and dysregulates cellular metabolism. Proc Natl Acad Sci U S A 2017;114:746–51. 28069950 10.1073/pnas.1611113114 PMC5278463

[50] Bozulic L, Surucu B, Hynx D, et al. PKBalpha/Akt1 acts downstream of DNA-PK in the DNA double-strand break response and promotes survival. Mol Cell 2008;30:203–13. 18439899 10.1016/j.molcel.2008.02.024

[51] Liu L, Dai X, Yin S, et al. DNA-PK promotes activation of the survival kinase AKT in response to DNA damage through an mTORC2-ECT2 pathway. Sci Signal 2022;15:eabh2290. 34982576 10.1126/scisignal.abh2290 PMC8992387

[52] Moller SH, Hsueh PC, Yu YR, et al. Metabolic programs tailor T cell immunity in viral infection, cancer, and aging. Cell Metab 2022;34:378–95. 35235773 10.1016/j.cmet.2022.02.003

[53] Yang Z et al. Restoring oxidant signaling suppresses proarthritogenic T cell effector functions in rheumatoid arthritis. Sci Transl Med 2016;8:331ra338. 10.1126/scitranslmed.aad7151 PMC5074090 27009267

[54] Shen Y, Wen Z, Li Y, et al. Metabolic control of the scaffold protein TKS5 in tissue-invasive, proinflammatory T cells. Nat Immunol 2017;18:1025–34. 28737753 10.1038/ni.3808 PMC5568495

[55] Telang S, Clem BF, Klarer Alden C, et al. Small molecule inhibition of 6-phosphofructo-2-kinase suppresses t cell activation. J Transl Med 2012;10:95. 22591674 10.1186/1479-5876-10-95 PMC3441391

[56] Choi SC, Titov AA, Abboud G, et al. Inhibition of glucose metabolism selectively targets autoreactive follicular helper T cells. Nat Commun 2018;9:4369. 30348969 10.1038/s41467-018-06686-0 PMC6197193

