# Supplementary material for: Cytoplasmic DNA sensing boosts CD4+ T cell metabolism for inflammatory induction
Source: Life Med. 2023 Jun 13;2(3):lnad021. doi: 10.1093/lifemedi/lnad021 (PMC11749111; doi:10.1093/lifemedi/lnad021)
Supplement: lnad021_suppl_Supplementary_Material [file lnad021_suppl_Supplementary_Material.docx]

**Supplementary information**

**Cytoplasmic DNA sensing boosts CD4^+^ T cell metabolism**

**for inflammatory induction**

by Ye et al.


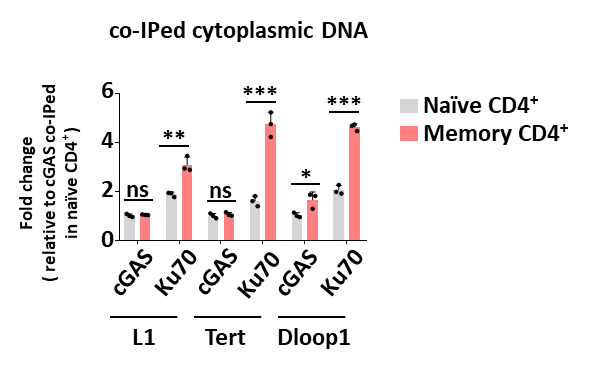


**Figure S1. DNA is sensed in CD4^+^ T cells through KU complex.**

qPCR analysis of co-Immunoprecipitated DNA with cGAS and KU70 in naïve CD4^+^ (CD44^-^CD62L^+^CD4^+^) and memory CD4^+^ (CD44^+^CD62L^-^CD4^+^) T cells *ex vivo*. Statistics, two-tailed Student’s *t* test. Error bars represent SD. Differences were considered to be significant at *p* < 0.05 and are indicated by *, those at *p* < 0.01 are indicated by **, and those at *p* < 0.001 are indicated by ***.

**
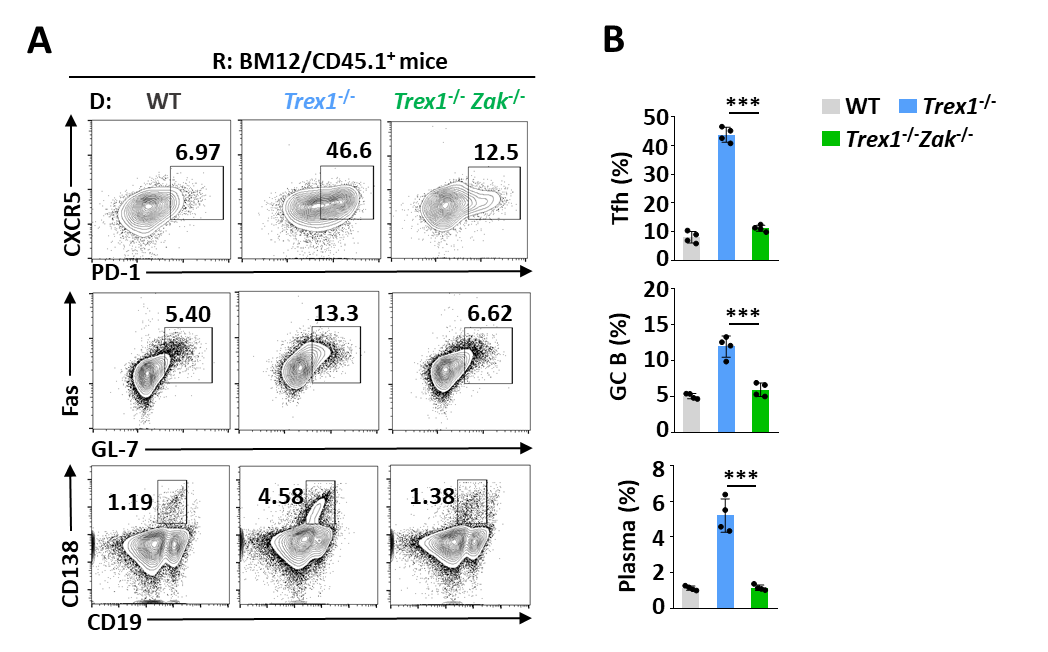
**

**Figure S2. *Zak* deficiency in CD4^+^ T cells abolishes DNA-boosted generation of Tfh, GC-B and plasma cells.**

**(A, B)** Flow cytometric analysis of Tfh (CXCR5^+^PD1^+^CD4^+^), GC B (Fas^+^GL-7^+^CD19^+^), and plasma cells (CD138^+^CD19^lo^) in the spleen. Data are presented as the representative FACS histograms (**A**) and summary graphs (**B**). Statistics, two-tailed Student’s *t* test. Error bars represent SD. Differences were considered to be significant at *p* < 0.05 and are indicated by *, those at *p* < 0.01 are indicated by **, and those at *p* < 0.001 are indicated by ***.**
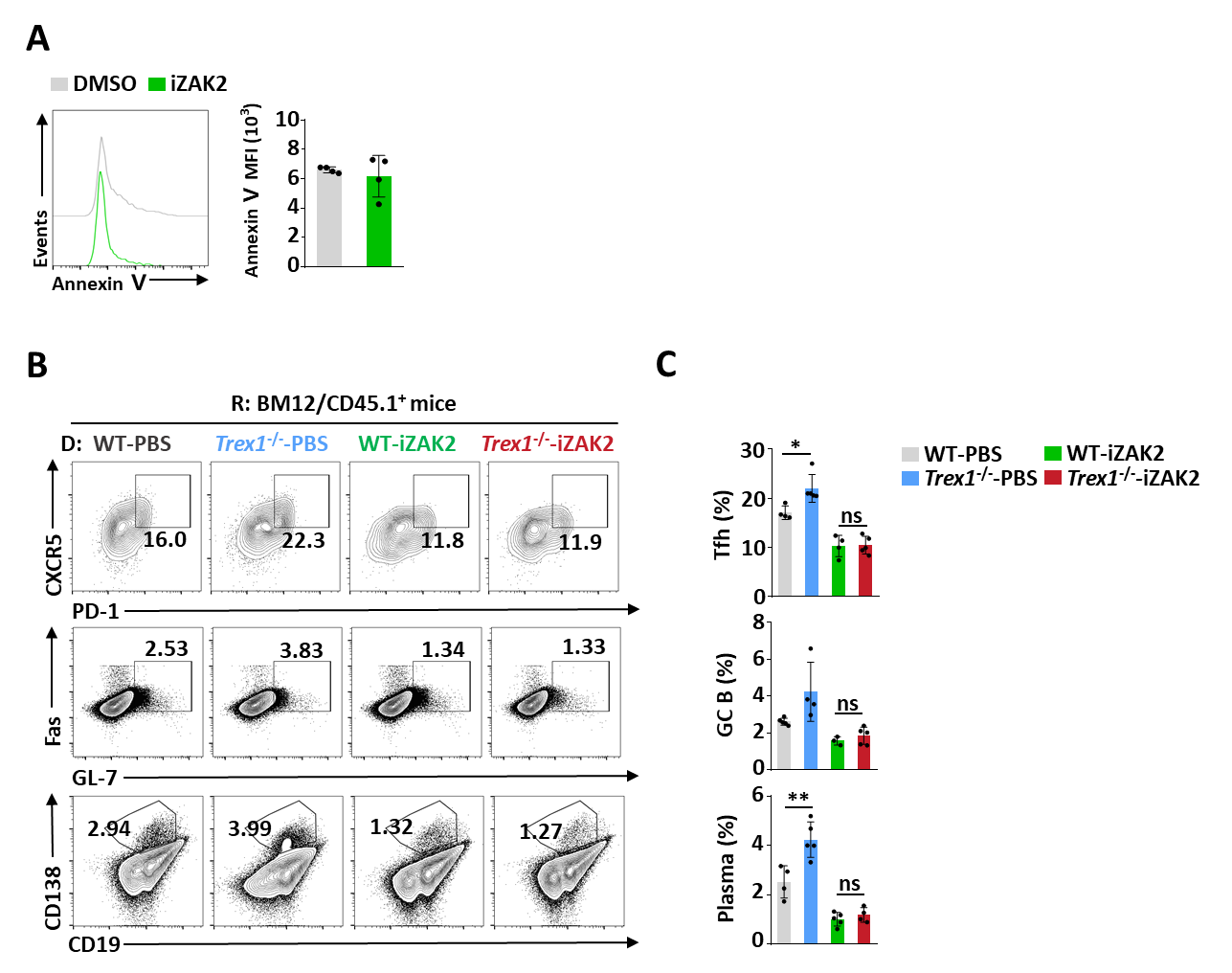
**

**Figure S3. Targeting ZAK inhibits DNA-boosted generation of Tfh, GC-B and plasma cells.**

1. Apoptosis analysis of CD4^+^ T cells treated with iZAK2 (1 μM) or DMSO as vehicle. Data are presented as the representative FACS histograms (**left**) and summary graphs (**right**). **(B, C)** Flow cytometric analysis of Tfh (CXCR5^+^PD1^+^CD4^+^), GC B (Fas^+^GL-7^+^CD19^+^), and plasma cells (CD138^+^CD19^lo^) in the spleen. Data are presented as the representative FACS histograms (**B**) and summary graphs (**C**). Statistics, two-tailed Student’s *t* test. Error bars represent SD. Differences were considered to be significant at *p* < 0.05 and are indicated by *, those at *p* < 0.01 are indicated by **, and those at *p* < 0.001 are indicated by ***.

**Table S1. Primers used for real-time quantitative PCR**

| Genes | Forward primers (5'-3') | Reverse primers (5'-3') |
| --- | --- | --- |
| m*Glut1* | ACCATCTTGGAGCTGTTCCG | GCCTTCTCGAAGATGCTCGT |
| m*Glut3* | GGATTCGCCAAGATAGCGGA | AACGATGCCCAGCTGGTTTA |
| m*Hk2* | TGATCGCCTGCTTATTCACGG | AACCGCCTAGAAATCTCCAGA |
| m*Pgk1* | ATGTCGCTTTCCAACAAGCTG | GCTCCATTGTCCAAGCAGAAT |
| m*Pkm2* | GCCGCCTGGACATTGACTC | CCATGAGAGAAATTCAGCCGAG |
| m*Aldoa* | CGTGTGAATCCCTGCATTGG | CAGCCCCTGGGTAGTTGTC |
| m*Eno1* | TGCGTCCACTGGCATCTAC | CAGAGCAGGCGCAATAGTTTTA |
| m*L1* | TAGGAAATTAGTTTGAATAGGTGAGAGGGT | TCCAGAAGCTGTCAGGTTCTCTGGC |
| m*Tert* | CTAGCTCATGTGTCAAGACCCTCTT | GCCAGCACGTTTCTCTCGTT |
| mDloop1 | CCCTTCCCCATTTGGTCT | TGGTTTCACGGAGGATGG |
| m*Actin* | CGTGAAAAGATGACCCAGATCA | CACAGCCTGGATGGCTACGT |
